# Greater Reduction in Contralesional Hand Use After Frontoparietal Than Frontal Motor Cortex Lesions in *Macaca mulatta*

**DOI:** 10.3389/fnsys.2021.592235

**Published:** 2021-03-18

**Authors:** Warren G. Darling, Marc A. Pizzimenti, Diane L. Rotella, Jizhi Ge, Kimberly S. Stilwell-Morecraft, Robert J. Morecraft

**Affiliations:** ^1^Department of Health and Human Physiology, Motor Control Laboratory, The University of Iowa, Iowa City, IA, United States; ^2^Department of Anatomy and Cell Biology, Carver College of Medicine, The University of Iowa, Iowa City, IA, United States; ^3^Division of Basic Biomedical Sciences, Laboratory of Neurological Sciences, The University of South Dakota, Sanford School of Medicine, Vermillion, SD, United States

**Keywords:** reach, grasp, manipulation, brain injury, hand

## Abstract

We previously reported that rhesus monkeys recover spontaneous use of the more impaired (contralesional) hand following neurosurgical lesions to the arm/hand representations of primary motor cortex (M1) and lateral premotor cortex (LPMC) (F2 lesion) when tested for reduced use (RU) in a fine motor task allowing use of either hand. Recovery occurred without constraint of the less impaired hand and with occasional forced use of the more impaired hand, which was the preferred hand for use in fine motor tasks before the lesion. Here, we compared recovery of five F2 lesion cases in the same RU test to recovery after unilateral lesions of M1, LPMC, S1 and anterior portion of parietal cortex (F2P2 lesion – four cases). Average and highest %use of the contralesional hand in the RU task in F2 cases were twice that in F2P2 cases (*p* < 0.05). Recovery in the RU task was closely associated with volume and percentage of lesion to caudal (new) M1 (M1c) in both F2 and F2P2 lesion cases. One F2P2 case, with the largest M1c lesion and a large rostral somatosensory cortex (S1r) lesion developed severe contralesional hand non-use despite exhibiting some recovery of fine motor function initially. We conclude that the degree of reduced use of the contralesional hand is primarily related to the volume of M1c injury and that severe non-use requires extensive injury to M1c and S1r. Thus, assessing peri-Rolandic injury extent in stroke patients may have prognostic value for predicting susceptibility to RU and non-use in rehabilitation.

## Introduction

Learned non-use (LNU) is a clinical term that refers to a motor deficit after nervous system damage due to learned suppression of limb use, reinforced over time by poor quality movements (Taub, 1980; Wolf et al., 1989). Persistent non-use of the impaired hand has been demonstrated in monkeys after unilateral neurosurgical lesions of motor cortex and surrounding regions (Ogden and Franz, 1917) and following unilateral dorsal root deafferentation of one upper limb (Taub et al., 2006). Non-use has also been observed in patients with unilateral cerebral palsy (Crocker et al., 1997), traumatic brain injury (Wolf et al., 1989) and stroke-induced hemiplegia (Wolf et al., 1989, 2006; Taub et al., 1993). This non-use is considered learned because it persists either fully, or as reduced use (RU), even when the monkey or human is able to use the impaired hand when forced. For example, we have shown that following surgical lesions limited to motor cortex monkeys can use the more impaired (contralesional) hand within a few days to climb, or within a few weeks to grasp small food objects when forced to use that hand (e.g., Darling et al., 2016). However, when given a choice these monkeys primarily use the less impaired (ipsilesional) hand in a fine motor task, indicating a degree of persistent RU of the more impaired (contralesional) hand (Darling et al., 2010). Here, we define reduced use as the post-lesion percentage use of the more impaired hand in a fine motor task in which the monkey could choose which hand to use (RU task) compared to percentage use of the same hand in a pre-lesion test of hand preference (Nudo et al., 1992) in which the monkey also had the choice of which hand to use. Non-use is defined as no use of the more impaired hand in the RU task. Whether such non-use should be considered learned, due to lack of motivation to use the impaired hand, neglect or impaired control of that hand when associated with damage to cortical motor areas is questionable. Thus, we use the term non-use, rather than LNU, throughout this report.

The classical observation that sensory loss from dorsal rhizotomy can induce non-use of the limb for several months, unless overcome by greatly increased motivation to use the limb, shows that injury to motor nerves or CNS motor areas is not required to induce this clinical condition (Taub et al., 1977). However, the severe loss of afferent input likely has consequences for CNS sensorimotor integration areas because of probable massive loss of synaptic connections into these areas (Pons et al., 1991; Woods et al., 2000; Darian-Smith et al., 2013, 2014). Moreover, both subcortical processing (by spinal, brainstem and cerebellar areas) and cortical processing of somatosensory input is presumably lost or considerably altered after such lesions. Such subcortical processing of sensory input is likely to be very important for online control of well-learned movements. Thus, its loss following dorsal rhizotomy may be primarily responsible for impairment that leads to non-use because of poor control of common and simple everyday movements (e.g., feeding, grooming, etc.) despite relatively intact motor regions of the brain and spinal cord. In contrast, sensory processing by cortical areas of the parietal and frontal lobes is presumably important especially for learning of novel movements and perhaps for control of learned voluntary movements in novel situations, leading to a weaker contribution to non-use or reduced use following dorsal root injury.

Despite an historical account of non-use occurring after lesion of motor cortex and surrounding regions (Ogden and Franz, 1917), the role of cerebral cortical injury in developing non-use remains unclear. In the rhesus monkey model, we have previously shown that RU of the contralesional hand occurs for varying lengths of time and to varying degrees following unilateral surgical lesions of frontal lobe motor areas including primary motor cortex (M1), M1 + lateral premotor cortex (LPMC) and M1 + LPMC + supplementary motor cortex (M2 or SMC) (Darling et al., 2010, 2014). Although occasional forced use (i.e., motor testing) of the contralesional hand in these experiments probably stimulated greater use of that hand, we have also observed spontaneous recovery of impaired hand use in two monkeys without any forced use or constraint of the less impaired limb after lesions of M1 and M1 + LPMC (Darling et al., 2014). Interestingly, these monkeys demonstrated non-use over the first three post-lesion weeks as they never used the contralesional hand to reach to and pick up small food objects during that time (in testing allowing use of either hand) but could demonstrate use of the hand and digits in climbing and grasping large objects such as the cage bars. However, they then began regularly using the more impaired hand in motor testing sessions.

Impaired use of the upper limb contralateral to an injured cortical sensory processing area in the parietal lobe might also induce non-use. Although some investigations have shown that unilateral damage limited to posterior parietal cortex area 7 in monkeys does not have any lasting effects on accuracy of upper limb reaching movements to visual targets (Faughier-Grimaud et al., 1978), others have reported that lesions of areas 5 and 7 in monkeys produce lasting inaccuracies of reaches to visual targets that included misorientation of the digits (Lamotte and Acuna, 1978). Such effects may lead to development of RU, especially in rhesus monkeys as they are rather ambidextrous, as indicated by relatively low pre-lesion handedness indexes of many monkeys, and demonstrate similar skill with the two hands prior to lesion in our research (e.g., Darling et al., 2016) and in previous studies (e.g., Lehman, 1980). Thus, rhesus monkeys might switch to primarily using the ipsilesional (less affected) hand for most/all fine motor tasks after a lesion of brain structures that primarily control the contralesional (more affected) hand. Indeed, early work in monkeys showed that damage limited to the primary somatosensory cortex (S1) of the postcentral gyrus (areas 3, 1 and 2) induces deterioration in control of fine contralesional finger movements used in grasping and grooming lasting 2 weeks and a preference to use the ipsilesional hand post-lesion, whereas either hand was used pre-lesion for fine tasks (Kennard and Kessler, 1940). These fine motor deficits worsened when the lesion was increased to include posterior parietal areas 5, 7. Grosser movements such as climbing were also affected, but less so when elicited by sight of food or emotions. Unfortunately, none of the non-human primate studies noted above performed quantitative motor assessments to examine use of each hand in a fine motor task in which the animal had a choice of which hand to use. Interestingly, humans with parietal lobe damage also exhibit persistent impaired grasping, suggesting specific deficits in control of fine hand/digit movements that may promote non-use or RU, but again there were no fine motor assessments allowing choice of which hand to use in a fine motor task (Binkofski et al., 1998).

The purpose of the present work was to test the hypothesis that combined frontoparietal lesions (which commonly occur following middle cerebral artery infarction) affecting arm areas of M1, LPMC, S1 and anterior part of the superior parietal lobule (F2P2 lesions) would cause greater and longer duration non-use or reduced use of the contralesional hand than lesions limited to arm areas of M1 and LPMC (F2 lesion). We tested this hypothesis by comparing use of each hand in a task in which the monkeys could choose which hand to use (RU task) in four monkeys with F2P2 lesions and five monkeys with F2 lesions. We also tested whether severity of lesion to caudal M1 (M1c) in the rostral bank of the central sulcus (which contains neurons with monosynaptic connections onto spinal motoneurons – (Rathelot and Strick, 2009; Lemon, 2019) and to rostral S1 (S1r) in the caudal bank of the central sulcus would cause greater and longer duration non-use or RU. The relation between recovery in the RU task and recovery of manipulation skill of the contralesional hand in tasks that required its use for successful target acquisition was also studied.

## Materials and Methods

### Subjects

**FIGURE 1:**
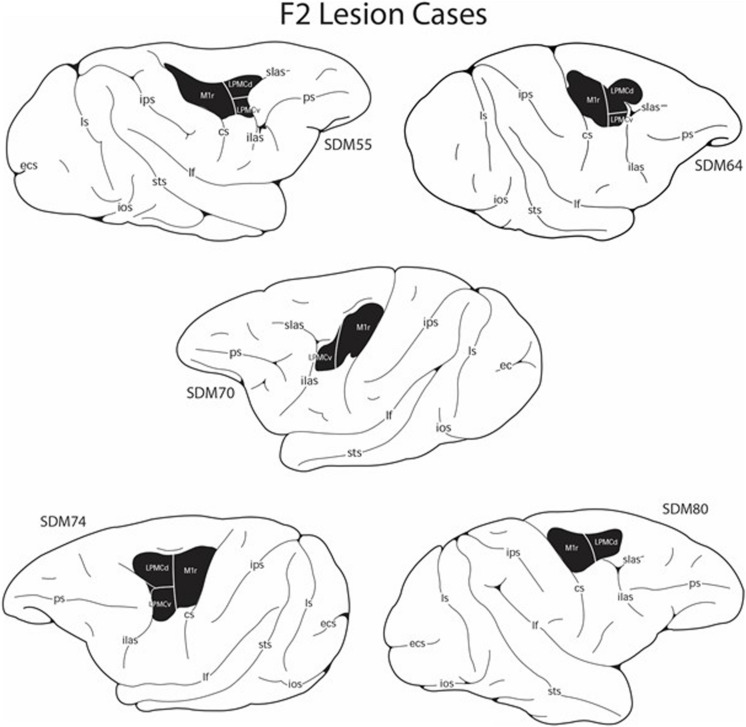
Line drawings of the lateral surface of the cerebral cortex showing the M1 + LPMC lesion site locations (blackened area) in the five F2 lesion cases. Detailed descriptions of the histological and cytoarchitectonic characteristics of each lesion are provided in previous reports along with microstimulation maps of the cortical surface that were used to guide the placement of each lesion (Darling et al., 2009; McNeal et al., 2010). cs, central sulcus; ecs, ectocalcarine sulcus; ilas, inferior limb of the arcuate sulcus; ios, inferior occipital sulcus; ips, intraparietal sulcus; lf, lateral fissure; ls, lunate sulcus; ots, occipito-temporal sulcus; ps, principal sulcus; slas, superior limb of the arcuate sulcus; sts, superior temporal sulcus.

**FIGURE 2:**
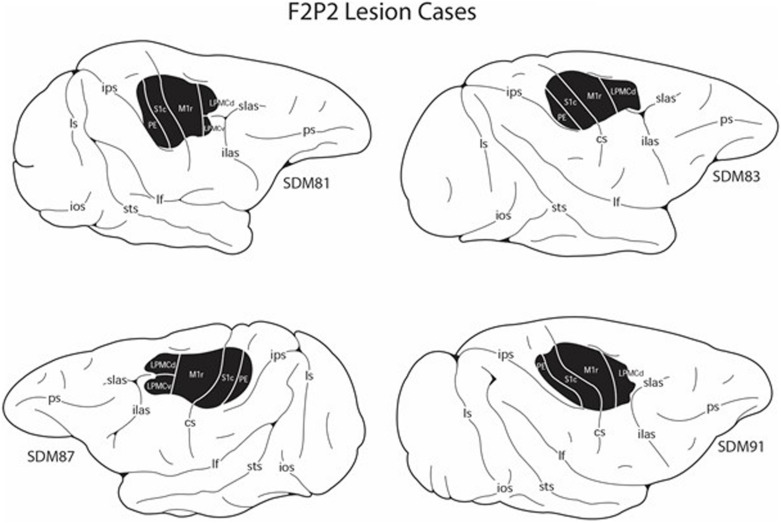
Line drawings of the lateral surface of the cerebral cortex showing the M1 + LPMC + anterior parietal cortex lesion site locations (blackened area) in the four F2P2 lesion cases. Detailed descriptions of the histological and cytoarchitectonic characteristics of each lesion are provided in previous reports along with microstimulation maps of the cortical surface that were used to guide the placement of each lesion (Morecraft et al., 2015; Darling et al., 2016). cs, central sulcus; ecs, ectocalcarine sulcus; ilas, inferior limb of the arcuate sulcus; ios, inferior occipital sulcus; ips, intraparietal sulcus; lf, lateral fissure; ls, lunate sulcus; ots, occipito-temporal sulcus; ps, principal sulcus; slas, superior limb of the arcuate sulcus; sts, superior temporal sulcus.

Nine monkeys (*Macaca mulatta*) served as subjects for these experiments (Table 1 and Figures 1, 2). Performance on reduced use tests and other motor tests in monkeys with lesions limited to frontal lobe motor areas (Table 1) were reported on previously (Darling et al., 2010, 2016). They are included here because they serve as an appropriate comparison group for the four monkeys with frontoparietal lesions that have also been reported on previously except for the RU testing. The monkeys were housed and maintained in a United States Department of Agriculture (USDA) approved and inspected facility. As described previously (Darling et al., 2010), all behavioral protocols were approved by the University of South Dakota Institutional Animal Care and Use Committee and performed according to United States Department of Agriculture, National Institutes of Health, and Society for Neuroscience guidelines for the ethical treatment of animals. Each monkey was evaluated by a primate veterinarian and judged to be healthy and free of neurological problems. One monkey (SDM55) had a physical defect in the third digit of the preferred hand, which was in a permanently extended position at the interphalangeal joints. However, this did not interfere with his ability to perform precision grasping/manipulation of small objects with digits one and two. To minimize possible training effects on manipulation, all monkeys had pre-lesion training/testing procedures for the motor tasks (e.g., to “learn” the task) but did not have fine digit training or fine digit cage enrichment toys after the lesion (i.e., toys which required use of dexterous movements – e.g., perforated ball, foraging boards with honey, etc.). However, visual and auditory enrichment programs were enhanced, daily contact with staff was increased and the animals were provided with toys and activities requiring proximal and gross motor activity.

**TABLE 1:** Characteristics and experimental parameters of monkeys.

**Case**	**Age^a^**	**Sex**	**HI^b^**	**Les. Cat.^c^**	**PLD^d^**	**GMLV^e^ (mm^3^)**		**M1cLV^f^ (mm^3^)%**		**S1rLV^g^ (mm^3^) %**		**WMLV^h^ (mm^3^)**	
**SDM**	**(years)**				**(months)**	**FL^i^**	**PL^j^**			**FL**	**PL**		
55	11.8	M	20L	F2	12	207.7	0.0	14.7	7.7	0.0	0.0	20.51	0.0
64	13.6	F	95.3L	F2	6	217.9	0.0	5.5	8.0	0.0	0.0	43.03	0.0
70	7.2	M	4.4R	F2	6	143.2	0.0	4.5	6.2	0.0	0.0	7.76	0.0
74	8.5	M	93.2R	F2	3	192.6	0.0	0.6	0.8	0.0	0.0	16.26	0.0
80	8.6	M	75.7L	F2	3	150.7	0.0	0.0	0.0	0.0	0.0	10.47	0.0
81	12	F	63.6L	F2P2	12	108.8	68.1	22.9	30.3	15.3	19.5	7.40	6.2
83	3.8	F	91L	F2P2	12	181.1	76.8	29.4	50.2	24.1	34.3	8.16	8.0
87	17	F	60R	F2P2	6	224.0	102.3	32.7	46.1	7.3	24.4	44.65	11.6
91	7.8	F	76L	F2P2	6	192.6	76.2	72.7	100.0	42.8	69.9	46.21	6.7

^*a*^Age at time of lesion; ^*b*^Handedness Index (percentage of initial reaches and retrievals with preferred hand – 50)*2 (Nudo et al., 1992); R, right hand preferred; L, left hand preferred; ^*c*^Lesion category – F2 (M1 + LPMC), F2P2 (M1 + LPMC + S1 + rostral area PE); ^*d*^PLD, post-lesion duration for recovery; ^*e*^GMLV, gray matter lesion volume; ^*f*^M1cLV, caudal M1 lesion volume in mm^3^ and as a percentage of total M1c volume; ^*g*^S1rLV, rostral S1 lesion volume in mm^3^ and as a percentage of total S1r volume; ^*h*^WMLV, white matter lesion volume; ^*i*^FL, frontal lobe; ^*j*^PL, parietal lobe.

### Experimental Apparatus

**FIGURE 3:**
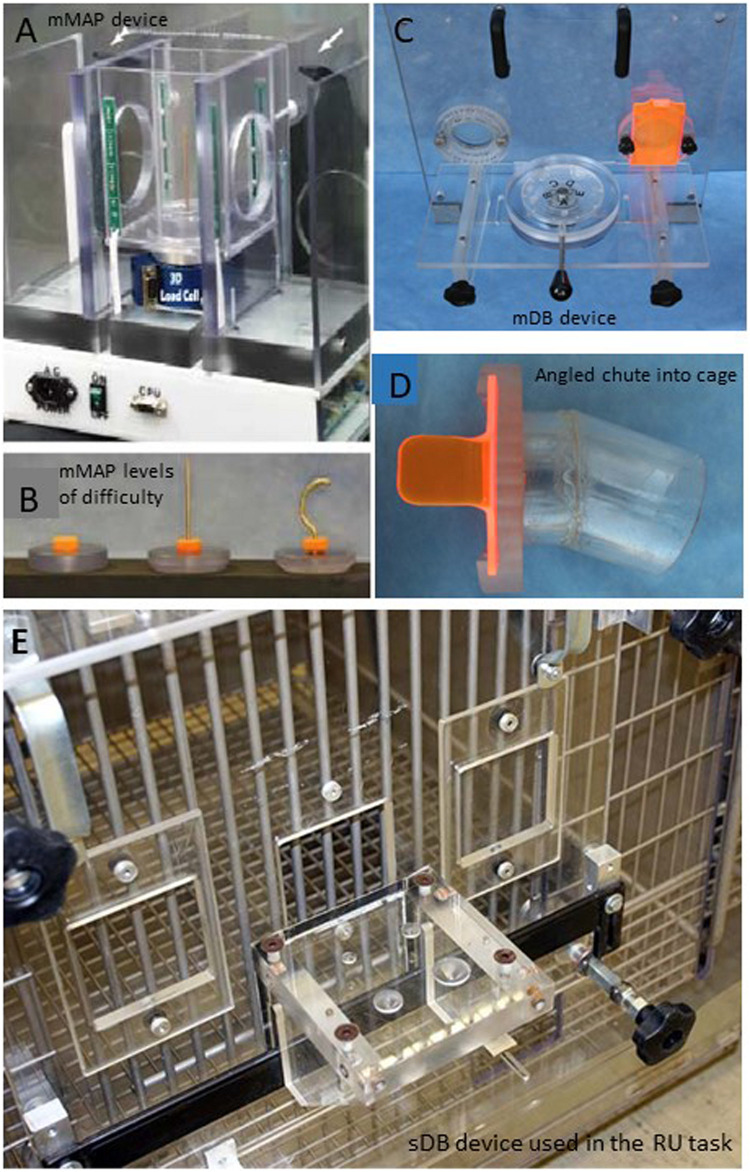
Pictures of the devices used to measure upper limb motor function. The modified movement assessment panel is shown in panel **(A)** with the different levels of difficulty shown below in panel **(B)**. The modified dexterity board is shown in panel **(C)** with the angled chute into the cage that the monkey has to move the hand through to reach to the food target. The angled chute constrains which hand can be used to successfully reach to the food target **(D)**. The standard dexterity board used in the pre-lesion handedness test and the post-lesion reduced use tests when the animals can choose which hand to use is shown in panel **(E)**.

Three devices were used for unimanual testing of fine hand motor performance in all monkeys. A fourth device was used on two of these monkeys (SDM74, SDM83) to test bimanual motor performance, but these findings are not reported in the current study. The unimanual testing devices used in this study included: (1) a standard dexterity board (sDB) to test for handedness before the lesion and for RU after the lesion (Figure 3E and Supplementary Video of RU task), (2) a modified dexterity board (mDB) to test for skill in forced use of the contralesional and ipsilesional hand to manipulate a small food pellet before and after the lesion (Figures 3C,D), 3) a modified movement assessment panel (mMAP) to test for skill in forced use of the contralesional and ipsilesional hand to manipulate a larger food object (carrot chip) before and after the lesion (Figures 3A,B). It should be noted that although the RU and handedness tasks use the same device (sDB), in the RU task the food pellets are placed on the flat surface of the sDB whereas in the test of handedness the food pellets are placed in the four wells of the sDB and on the flat surface as described previously (Darling et al., 2009).

Specifically, the sDB allowed the monkey to choose which hand to use to acquire small food pellets from four wells ranging in diameter from 13 to 25 mm and from its flat surface before the lesion in handedness testing. It was also used to test for RU or non-use after the lesion but with pellets placed only on the flat surface so that the pellet could be easily acquired by either hand. This device was also used to determine pre-lesion hand preference before any training began (on all the other tasks). The mDB (Pizzimenti et al., 2007) and mMAP (Darling et al., 2006) devices were used for motor testing of each hand before and after the lesion to assess functional recovery of hand/digit function skill. These two devices allow the experimenter to control which hand the monkey is able to use to acquire food targets. Specifically, this was accomplished by opening or closing right and left portal doors in addition to mechanical constraints placed on the hand path to allow for controlled testing of each hand without the need for restraints on one limb.

### Behavioral Procedures for Handedness Index, Reduced Use, and Motor Performance

Prior to all motor testing sessions, the monkey was food restricted for 18–24 h. Water was available at all times. Video recording of hand preference testing and spontaneous hand use post-lesion (with the sDB) was used to acquire data on the number of reaches with each hand to the food targets. During the pre-lesion hand preference testing sessions, the monkey had opportunities to retrieve 10 food pellets from each of the four wells and from the flat surface (50 pellets in total, random order of pellet placements) in three separate testing sessions (totaling 150 trials) were conducted for each animal. Data recorded included the number of reaches with each hand and number of successful acquisitions. These were used to compute a pre-lesion handedness index which reflects strength of hand preference (Nudo et al., 1992). During post-lesion RU testing, the food pellets were placed centrally on the flat surface of the sDB with either hand allowed to reach for pellet. We simply recorded the number of reaches with each hand over about 20 trials (note that if the monkey first reached with one hand and was unsuccessful and then reached with the other, it was counted as 2 reaches – one with each hand). We also recorded whether the monkey was successful in acquiring the food pellet. A high %use of the ipsilesional (less impaired) hand compared to its use in the pre-lesion handedness test was taken as evidence of reduced use of the contralesional (more impaired hand). Post-lesion testing began 1 week after the lesion and was carried out weekly for the first eight post-lesion weeks and every other week thereafter.

Full pre- and post-lesion testing sessions with the mDB included five retrieval attempts for each of the wells (A–E) for both limbs proceeding from the easiest well (E) to the most difficult (A), thereby giving the monkey 50 opportunities to retrieve pellets (25 with each hand). During post-lesion tests, the more impaired hand (contralateral to the surgically induced lesion) was always tested first to ensure high motivation. Full testing sessions with the mMAP included blocks of five trials at each difficulty level (i.e., flat surface, straight rod and curved rod) with each hand, thereby giving the monkey 30 opportunities to retrieve carrot chips (15 with each hand). Pre-lesion tests with the mDB and mMAP devices occurred approximately weekly with a minimum of six pre-lesion tests. Post-lesion testing with the mDB and mMAP devices was performed weekly for the first eight post-lesion weeks and then every other week for 6–12 months. The monkeys’ exposure to the mDB and mMAP tasks were limited to the experimental testing sessions and they had limited opportunities to use fine motor control at other times (e.g., only during feeding as there were no toys available to encourage fine movement control). Post-lesion RU tests involved 20 trials with the food pellets placed on the central part of the flat surface of the sDB and was carried out on the same schedule as the mDB and mMAP testing.

### Data Acquisition

Quantitative measurements of movement kinematics and kinetics were made in training sessions and testing sessions before the lesion and at regular intervals after the lesion using the mDB and mMAP devices as described previously to assess pre-lesion performance and post-lesion recovery in the mDB and mMAP tasks (Darling et al., 2006, 2009; Pizzimenti et al., 2007; McNeal et al., 2010). Handedness testing was performed only in the pre-lesion phase using the sDB. RU testing was performed only in the post-lesion phase using the sDB.

### Surgical Procedure

An overview of these procedures is described briefly here as they have been detailed previously (Pizzimenti et al., 2007; Darling et al., 2009; McNeal et al., 2010; Morecraft et al., 2015). All lesions were made in the cortical hemisphere contralateral to the preferred limb (as determined from pre-lesion hand preference score). The planned surgical lesions included the arm areas of M1 + the adjacent LPMC (category F2 lesion – Figure 1) and M1 + LPMC + S1 + anterior part of posterior parietal cortex (category F2P2 lesion – Figure 2). These lesions were studied because they partially simulate the damage experienced in middle cerebral artery cortical stroke and allow comparison of the effects of pure frontal lobe motor area injury to a similar lesion with the addition of damage to sensory processing areas of the parietal lobe. These experiments were also conducted to study the effects of these lesions on plasticity of corticobulbar and corticospinal terminations involved in control of hand movement (McNeal et al., 2010; Morecraft et al., 2015, 2016; Darling et al., 2018).

After aseptic cortical exposure under isoflurane anesthesia, the animal was transferred to intravenous ketamine anesthesia and intracortical microstimulation (ICMS) was used to localize the arm areas of M1, LPMC and, in F2P2 cases, anterior parietal cortex (Morecraft et al., 2001, 2002, 2007, 2015, 2016; McNeal et al., 2010). The animal was transferred back to isoflurane anesthesia immediately after ICMS mapping. Cortical vessels supplying the arm areas to be lesioned were then cauterized. Following a 5- to 10-min waiting period, gray matter of the arm area(s) was removed using subpial aspiration. The dura was then sutured closed, the bone flap replaced and anchored to the cranium and the skin closed with sterile sutures. As detailed in our previous papers, pre- (24 h before surgery) and post-surgical (9–12 days) antibiotics were administered including postsurgical analgesics for 48–72 h. In all cases, a second neurosurgery was performed 33–34 days prior to sacrifice to inject neural tract tracers into arm areas of spared, intact motor cortices. As noted, these tract tracing experiments were designed to investigate neuroplasticity in intact neuronal projection systems and associated results have been reported (McNeal et al., 2010; Morecraft et al., 2015, 2016; Darling et al., 2018). Complete anatomical descriptions of the lesions in all these monkeys are presented in previous studies (Darling et al., 2009, 2016; McNeal et al., 2010; Morecraft et al., 2015, 2016).

### Estimation of Lesion Volume

Lesion volumes were estimated as described previously by examining damage to Nissl stained tissue sections at 500 μm intervals through the lesion site (Darling et al., 2009, 2016; McNeal et al., 2010). Effects of post-surgical atrophic distortions were minimized by superimposing an outline of the lesion site onto the contralateral undamaged hemisphere. Lesion volume of caudal M1c, which contains many neurons with monosynaptic connections onto lamina IX motoneurons, was estimated because of its probable importance in control of fine hand/digit movements (Lemon and Griffiths, 2005; Rathelot and Strick, 2006, 2009; Lemon, 2008). Lesion volume of S1r was also estimated because it is the main entry point for somatosensory inputs to cerebral cortex (Kaas, 2012). Also, short somatosensory projections from S1r neurons to M1c neurons may be very important in fine motor control (Darian-Smith et al., 1993; Kaneko et al., 1994a,b).

We also estimated percentage of M1c and S1r arm/hand area that was lesioned. These were computed based on total gray matter lesion volume in that area relative to total volume of that area defined by ICMS and post-mortem cytoarchitectural analysis (Darling et al., 2016). In the parietal lobe, rostral S1 is the cortex lining the caudal bank of the central sulcus (see Figures 3, 4 in Darling et al., 2016). Cytoarchitectonically this corresponds to areas 3a and 3b and that part of area 1 that lines the upper region of the caudal bank cortex (Morecraft et al., 2004). Caudal S1 is on the gyral surface of the parietal lobe and cytoarchitecturally corresponds to the gyral portion of area 1 and the adjacent area 2. In the frontal lobe, the caudal border of M1c is sharply defined at the rostral border (termination) of area 3a of S1r, in the fundus of the central sulcus (see Figures 3, 4 in Darling et al., 2016). M1c continues from this point as the cortex lining the rostral bank of the central sulcus which corresponds to area 4. Cytoarchitectonically, area 4 is identified in part by the presence of large Betz cells in layer V and the lack of granular layers II and IV (Morecraft et al., 2012). The cortex of M1c ends on the central sulcus convexity. M1r lies is rostral to this point and corresponds to area 4 on the cortical surface. LPMC (area 6) is rostral to M1r/area 4.

**FIGURE 4:**
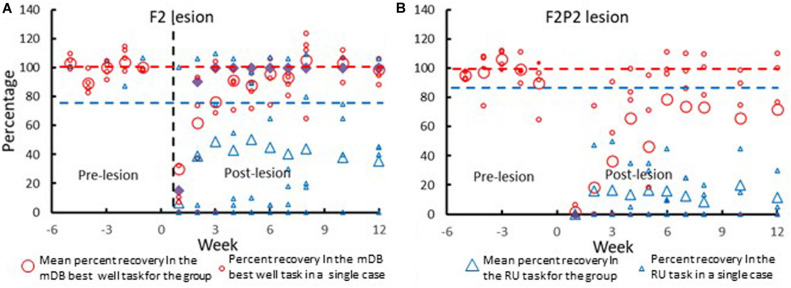
Recovery of contralesional hand performance in the mDB and RU tasks in all F2 **(A)** and F2P2 **(B)** lesion cases. The large symbols show mean data across all subjects within the group. The smaller symbols show data from individual monkeys. Recovery of performance in the mDB best well task (red circles) was computed as the percentage ratio of post-lesion performance score/average prelesion performance score (over the last five pre-lesion tests) on the well with highest pre-lesion skill (best well). Recovery of spontaneous use of the contralesional hand in the RU task (blue triangles) is plotted as the %use of that hand in the post-lesion tests. The red dashed horizontal line represents average pre-lesion performance scores over the last five pre-lesion tests. The dashed blue horizontal line represents pre-lesion use of the preferred hand in the test of hand preference. Note that data for the RU task for SDM91 of the F2P2 lesion group was all zeros as this monkey never use the contralesional hand in the RU task. Similarly, SDM91 had very poor recovery in the mDB best well task as there were no attempts made to reach to and grasp the food target (performance score = 0) on most trials.

### Data Analysis

Manipulation performance scores on individual trials in each pre- and post-lesion mDB and mMAP testing session and skill in manipulation of the food targets were computed as described previously (Pizzimenti et al., 2007; Darling et al., 2009). Briefly, applied force, duration and outcome data were used to compute the manipulation performance scores on each trial in the most difficult mMAP (curved rod) task (Figures 3A,B; equations 1 and 2). Manipulation performance scores were computed on each trial in the mDB (Figures 3C,D) best well (with the highest pre-lesion skill over the last five pre-lesion test) and a 2^nd^ smaller well (with pre-lesion skill about 1/2 that of the best well) based on outcome, manipulation duration, number of times contact was lost between the digit and food target as described in equation 3. Pre-lesion manipulation skill was computed as the mean of manipulation performance scores divided by the S.D. of manipulation performance scores over the last five testing sessions (25 trials) before the lesion. The maximum post-lesion manipulation skill was computed as the highest manipulation skill over five consecutive testing sessions during the first 12 weeks of post-lesion testing. Recovery of manipulation skill was defined as the ratio of post-lesion maximum manipulation skill divided by pre-lesion manipulation skill. We used maximum post-lesion skill because this measure provides the best estimate of potential for recovery of skill.

(1) $$\mathrm{TAImp}\,\left(n\right)=\int \left|F_{x}\right|\,\mathrm{dt}+\left|F_{y}\right|\,\mathrm{dt}+\left|F_{z}\right|\,\mathrm{dt}$$

TAImp⁢(n)-total absolute impulse of trial n

∫-integral over duration of trial t with respect to time (dt)

$$F_{x}-\text{Force applied in left/right direction}$$

$$F_{y}-\text{Force applied in anterior-posterior direction}$$

$$F_{z}-\text{Force applied in vertical direction}$$

If outcome ≥ 2 (i.e., successful grasp and lift/manipulation of the carrot chip) then

$${\mathrm{PS}}_{\mathrm{mMAP}}\left(n\right)=\{100*(\left(\mathrm{MaxTAImp}-\mathrm{TAImp}\left(n\right)\right)/\mathrm{TAImp}\mathrm{Range})+100*\left(\left(\mathrm{MaxDur}-\mathrm{Dur}\left(n\right)\right)/\mathrm{DurRange}\right)\}*\mathrm{Outcome}\,\left(n\right)$$

(2) $$\mathrm{If}\,{\mathrm{PS}}_{\mathrm{mMAP}}\,\left(n\right)<200\,\mathrm{then}\,{\mathrm{PS}}_{\mathrm{mMAP}}\,\left(n\right)=200$$

Else

$${\mathrm{PS}}_{\mathrm{mMAP}}\left(n\right)=\{100*(\left(\mathrm{TAImp}\left(n\right)-\mathrm{MinTAImp}\right)/\mathrm{TAImp}\mathrm{range})+100*(\left(\mathrm{Dur}\left(n\right)-\mathrm{MinDur}\right)/\mathrm{DurRange})\}*\mathrm{Outcome}(n)$$

$$\mathrm{If}\,{\mathrm{PS}}_{\mathrm{mMAAP}}\,\left(n_{j}\right)>200\,\mathrm{then}\,{\mathrm{PS}}_{\mathrm{mMAP}}\,\left(n\right)=200$$

$$\mathrm{If}\,{\mathrm{PS}}_{\mathrm{mMAAP}}\,\left(n_{j}\right)<50\,\mathrm{then}\,{\mathrm{PS}}_{\mathrm{mMAP}}\,\left(n\right)=50$$

Where:

PS_mMAP_(n) – performance score on mMAP trial n
Outcome(n) – success on trial n (0 for no attempt with the correct hand, 1 for unsuccessful attempt with the correct hand, 2 if the carrot chip is successfully grasped and lifted over the rod but then dropped and not removed from the food chamber, 3 if the carrot chip is successfully grasped and lifted over the rod but then dropped and removed from the food chamber, 4 for successful acquisition without dropping the carrot chip)
MinTAImp – minimum single trial pre-lesion total absolute impulse within a difficulty level for either hand
MaxTAImp – maximum single trial pre-lesion total absolute impulse within a difficulty level for either hand
TAImp Range – MaxTAImp – MinTAImp
Dur(n) – duration of trial n
MinDur – minimum single trial duration during pre-lesion tests with either hand within a difficulty level
MaxDur – maximum single trial duration during pre-lesion tests with either hand within a difficulty level
DurRange – MaxDur – MinDur

(3) MS⁢(n)=m⁢(n)×[100×(Mdur+C)]

Where:

MS_(n)_ is manipulation score of trial n
m_(n)_ is the multiplier (0 for no attempt, 1 for failure, 2 for successful retrieval of the pellet)

$$\text{Mdur}=\frac{\left(\begin{array}{c} \mathrm{maximum}\,\mathrm{prelesion}\,\mathrm{manipulation}\,\mathrm{duration}\\ -\mathrm{manipulation}\,\mathrm{duration}\,\mathrm{on}\,\mathrm{trial}\,n \end{array}\right)}{\left(\begin{array}{c} \text{maximum}-\mathrm{minimum}\,\mathrm{prelesion}\\ \mathrm{manipulation}\,\mathrm{duration} \end{array}\right)}$$

C = 1/(1 + number of times contact is broken between a digit and the pellet on trial n)

Data compiled from video recordings of the post-lesion RU tests for the contralesional (more impaired) hand included: (1) week of first attempt, (2) week of first successful acquisition, and (3) %use the impaired hand in each RU test over 3 months of post-lesion recovery because of the variation in post-lesion duration for recovery (Table 1). For comparison, we obtained from the post-lesion mDB testing sessions for the contralesional hand the week of: (1) first attempt on any well, (2) first successful acquisition on any well and (3) first test session on which there were successful acquisitions on all five trials on the well with highest pre-lesion skill and a 2^nd^ smaller well with lower skill (see above for computation of skill). Similarly, we obtained from the mMAP curved rod task testing the week of: (1) first attempt, (2) first successful acquisition and (3) first testing session with successful acquisitions on all five trials. To test whether recovery in the RU task differed between the two groups we used independent *t*-tests comparing average %use of the more impaired hand in the RU tests after the hand was first used in the RU tests and the highest %use of that hand in a single RU test during the first three post-lesion months. Exploratory (no corrections for multiple tests of significance) single linear regression analyses were used to examine whether use of the more impaired hand on the non-use tests was associated with recovery of its manipulation skill in the mDB and mMAP tests and on volume of gray and white matter lesions.

## Results

Detailed descriptions of the anatomy of the frontal and frontoparietal lesions of all these animals as well as effects of these lesions on upper limb motor function have been provided previously (Darling et al., 2009, 2016; McNeal et al., 2010; Morecraft et al., 2015). Briefly, after the lesion surgery there was a period of paresis of the contralesional hand for postural support and other motor tasks in all monkeys that lasted a few days. However, compared to cases with F2 lesions, notable differences in F2P2 cases which persisted throughout the post-lesion survival/testing period included: (1) dragging of the contralesional hand with finger-tips contacting the cage floor during quadrupedal gait, and (2) attentively looking at the hand after picking up food in motor tests. We attribute these behaviors to the animals being unaware of tactile inputs from the impaired hand. Recovery of contralesional upper limb function in both F2 and F2P2 lesioned cases began during the first post-lesion week in most cases as indicated by attempts and/or successful acquisitions of small food targets in the mDB and mMAP tasks at the 1^st^ post-lesion motor testing 1 week after the lesion (see Table 2 of Darling et al., 2016). However, F2P2 lesioned monkeys usually showed slower and poorer recovery of both hands than F2 lesioned monkeys on the mDB and mMAP motor tests as described previously (Morecraft et al., 2015; Darling et al., 2016, 2020). One F2P2 lesion case (SDM91) with lesions estimated to include all of M1c and 69.9% of S1r developed non-use of the contralesional hand in motor tests after having consistent success in both the mDB task (on wells 19 mm or more in diameter) and on the easiest mMAP task (acquiring the carrot chip from a flat surface) during the first eight post-lesion weeks (Darling et al., 2016).

**TABLE 2:** Initial recovery in the mDB, mMAP, and RU tests and manipulation skill recovery for each case.

**Case**	**Post-lesion week of 1^st^ attempt**			**Post-lesion week of 1^st^ success**			**Post-lesion week of All success**		
**SDM**	**mDB AW^a^**	**mMAP CR^b^**	**RU**	**mDB AW**	**mMAP CR**	**RU**	**mDB BW^c^**	**mDB W2^d^**	** MAP CR**
55	1	2	2	2	4	7	4	4	5
64	4	4	6	4	7	6	6	5	8
70	1	1	4	1	1	4	2	4	2
74	1	1	1	1	1	1	1	1	1
80	1	2	2	2	2	2	2	2	2
**Mean F2 cases**	**1.6**	**2**	**3**	**2**	**3**	**4**	**3**	**3.6**	**3.6**
81	1	1	4	2	4	4	6^e^	6	3
83	1	2	2	3	7	2	3	NS^f^	2
87	3	5	NA^g^	3	4	NA	4	6	6
91	4	4	na^h^	5	na	NS	na		
**Mean F2P2 cases**	**2.3**	**3**	**3^i^**	**3.3**	**5^i^**	**3^i^**	**4.3^+^**	**5^i^**	**3.7^i^**

*^*a*^Any well; ^*b*^Curved rod task; ^*c*^Well with highest skill; ^*d*^Smaller well with skill of about 50% of skill on best well; ^*e*^SDM81 often dropped the food pellet in the best well (E – a small dimple to hold the food pellet) task during the post-lesion phase, presumably due to lack of an adequate grasp and did not have consistent success. We thus used the post-lesion week of all success on any well rather than the best well; ^*f*^NS, never successful on all trials in a single test session in this test; ^*g*^NA, not available (1^*st*^ post-lesion RU test was at 5^*th*^ post-lesion week when SDM87 used the contralesional hand to successfully acquire the food pellet on 35% of trials); ^*h*^na, no attempts with the contralesional hand on the RU task during the entire post-lesion period; ^*i*^Mean values computed without including cases with NS, NA, na.*

**FIGURE 5:**
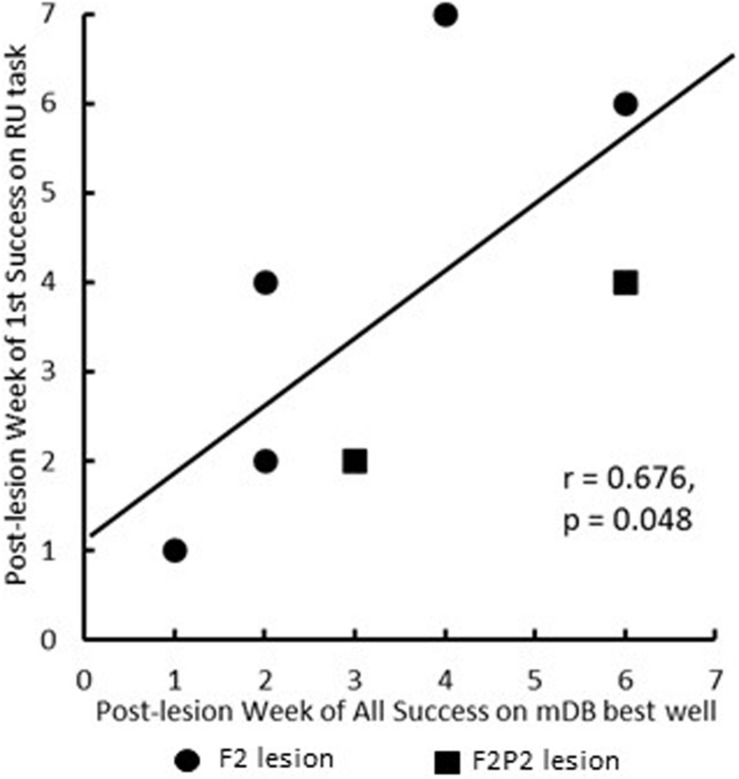
Scattergraph showing the relationship between post-lesion week of 1^st^ success on the RU task and post-lesion week of consistent success (on all five trials) of the mDB best well task. Each plotted point is for a single monkey. Note that the graph does not include one F2P2 case (SDM91) that never attempted to use the contralesional hand in the RU task and another F2P2 case (SDM87) was successful in its first RU test that occurred at 5 weeks post-lesion.

Recovery in the mDB, mMAP and RU motor tests occurred on average sooner and better in the F2 than in the F2P2 lesion cases (Figure 4 and Tables 2, 3). Recovery in the RU task clearly began sooner and to higher %use for F2 cases than for F2P2 cases (Figure 4 – blue triangles). Most F2 cases (3 of 5) made their first attempt with the contralesional hand in the RU test at the 1^st^ or 2^nd^ post-lesion week testing session whereas only one F2P2 case (SDM83) attempted by the 2^nd^ post-lesion week and another (SDM91) never used the contralesional hand in the RU task (Figure 4 and Table 2). Although SDM91 exhibited persistent non-use of the contralesional hand in the RU task, this case was able to successfully acquire food pellets in the larger wells of the mDB task using precision grasp during post-lesion weeks 3–8 (Morecraft et al., 2015), indicating possible LNU after post-lesion week 8. The first success in the RU task with the contralesional hand occurred in most cases at least 1 week after the first success in the mDB task, but one F2P2 lesion case (SDM83) was successful in the RU task 1 week before achieving success in the mDB task (Table 2). However, only two F2 lesion cases and one F2P2 lesion case successfully acquired the food pellet with the contralesional hand in the RU test in the 1^st^ or 2^nd^ post-lesion week (Table 2). Consistent success on all trials in the mDB and mMAP tasks in a single testing session was also achieved earlier in the F2 lesion cases (Table 2). Indeed, two of the F2P2 lesion cases never achieved consistent success on the smaller 2^nd^ well of the mDB task and one (SDM91) was never successful in the mMAP curved rod task. In contrast, all the F2 lesion monkeys achieved consistent success in the mDB and mMAP tasks (Table 2). The first successful use of the more impaired hand in the RU task usually occurred in the same post-lesion week or after the post-lesion week of a successful acquisition in the mDB best well or mMAP curved rod tasks (Table 2). The post-lesion week of 1^st^ success on the RU task was positively correlated with post-lesion week of consistent success on the mDB best well task (Figure 5) but not with post-lesion week of 1^st^ success on any well in the mDB task or the mMAP curved rod task (*p* > 0.05). Note, however, that post-lesion week of consistent success on the mDB task occurred after post-lesion week of first success on the RU task in two F2 lesion cases and two F2P2 lesion cases. Thus, success in the RU task could precede consistent success in the mDB best well task.

**TABLE 3:** %use of the contralesional hand in the RU task and recovery of manipulation skill during the first 12 post-lesion weeks.

**Case**	**%use in handedness test (HT)**	**Avg. %use after 1^st^ use during 1^st^ 12 weeks post-lesion^a^ (diff. from %use in HT)^b^**	**Highest %use in 1^st^ 12 weeks post-lesion^c^ (diff. from %use in HT)**	**Highest %use observed^d^ (diff. from %use in HT)**	**Manip skill recovery ratio^e^**		
					**mDB BW**	**W2**	**mMAP CR**
55	60	18.6 (−41.4)	75 (15)	100 (40)	0.80	1.22	0.76
64	97.7	8.4 (−89.3)	45.5 (−52.2)	45.5 (−52.2)	0.88	0.81	0.34
70	52.2	9.1 (−43.1)	40 (−12.2)	40 (−12.2)	1.03	1.28	0.95
74	96.6	91.4 (−5.2)	100 (3.4)	100 (3.4)	7.30	0.70	1.03
80	87.9	42 (−45.9)	89.5 (1.6)	89.5 (1.6)	0.72	0.72	2.34
**Mean F2 cases**	**78.9**	**33.9 (−45)**	**70 (−8.9)**	**75 (−3.9)**	**2.15**	**0.95**	**1.08**
81	81.8	3.3 (−78.5)	9.5 (−72.3)	20 (−61.8)	0.53	1.03	0.5
83	95.5	32.3 (−63.2)	50 (−45.5)	55 (−40.5)	0.72	0.31	0.91
87	80	27 (−53)	35 (−45)	45 (−35)	0.90	0.39	0.55
91	88	0 (−88)	0 (−88)	0 (−88)	0.05	0.00	0.06
**Mean F2P2 cases**	**86.3**	**15.6 (−70.7)**	26.1* (−62.7)	30* (−56.3)	**0.55**	**0.44**	**0.51**

^*a*^Average %use of the contralesional hand in RU tests beginning from test session with 1^*st*^ use of that hand in RU testing. ^*b*^%use in RU test – %use in pre-lesion hand preference test. ^*c*^Highest %use of the contralesional hand in a single RU test during the first 12 post-lesion weeks. ^*d*^Highest %use of the contralesional hand in a single RU tests during the entire post-lesion period. ^*e*^Highest manipulation skill recovery ratio during first 12 post-lesion weeks. *p < 0.05 for *t*-test comparison of %use of the contralesional hand in F2 cases and F2P2 cases.

**FIGURE 6:**
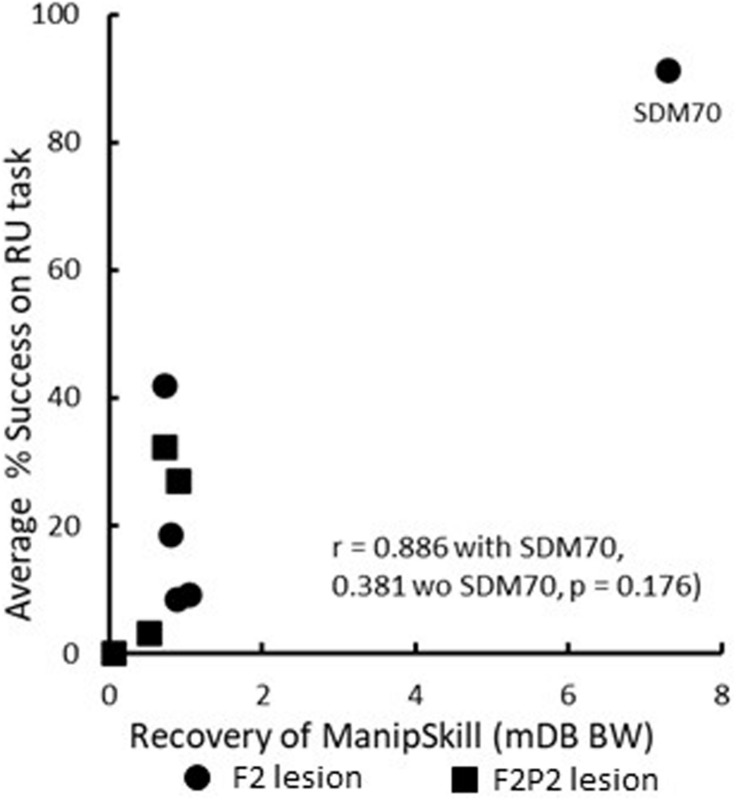
Scattergraph showing the relationship between average percentage use of the contralesional hand in the RU task after the first attempt and recovery of manipulation skill in the mDB best well (BW) task. Each plotted point is for a single monkey. Note that SDM70 had a very high manipulation skill recovery ratio that was primarily due to very low variability in performance scores. This monkey also had a very high average percentage success in the RU task, resulting in a spuriously high correlation with recovery of manipulation skill.

**FIGURE 7:**
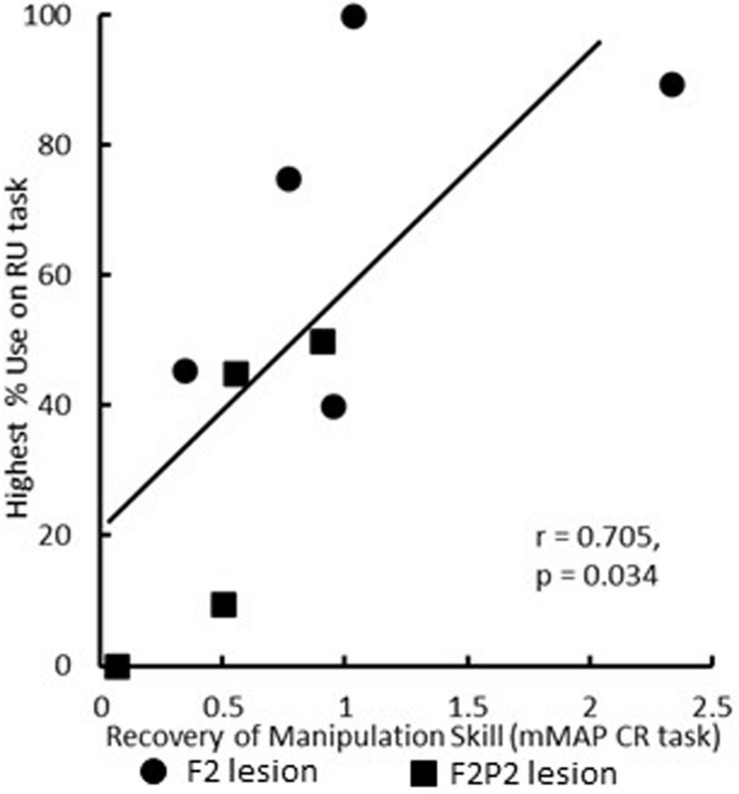
Scattergraph showing the relationship between highest percentage use of the contralesional hand in a single RU test and recovery of manipulation skill in the mMAP curved rod (CR) task. Each plotted point is for a single monkey. All nine monkeys were included in this figure and correlation analysis.

Recovery in the RU task (i.e., toward similar %use of the contralesional hand in the RU task as in the pre-lesion handedness test completed prior to the lesion) and of manipulation skill in the mDB and mMAP tasks was faster and to higher levels of skill in the F2 than in the F2P2 lesion cases, but there was considerable inter-subject variability in both groups (Table 3). Over the first 12 post-lesion weeks, %use of the contralesional hand in the RU task after its first use in that task (indicating motivation to use that hand) averaged 45% and 70.7% lower than %use of that hand in the pre-lesion handedness test in the F2 and F2P2 cases respectively (Table 3). F2 lesion cases averaged about double the %use in the RU task of F2P2 lesion cases, but there was high inter-subject variability in both groups resulting in no statistical differences between groups (Table 3, *p* = 0.371). Average %use of the contralesional hand in the RU task appeared to be strongly correlated with recovery of manipulation skill in the mDB best well task. However, this was a spurious correlation due primarily to SDM 70 (F2 lesion) as this case had high use of contralesional hand in the RU task and a very high recovery of manipulation skill in the mDB best well task (Figure 6). The highest %use of the contralesional hand in a single RU test session during the first 12 post-lesion weeks averaged 2.7X higher in F2 than in F2P2 lesion cases (Table 3, *p* = 0.047). Indeed, the highest %use of three F2 lesion cases exceeded the %use in the pre-lesion handedness test (Table 3). In contrast, the F2P2 cases all used the pre-lesion preferred hand far below its %use in the pre-lesion handedness test (Table 3). Consistent with these findings, recovery of manipulation skill in the mDB and mMAP tasks during the first 12 post-lesion weeks also averaged better in F2 than in F2P2 lesion cases, although again there was high inter-subject variability in both groups and no statistical differences (Table 3, *p* > 0.066). Finally, the highest %use of the contralesional hand in a single session of the RU test was positively correlated with recovery of manipulation skill on the mMAP curved rod task in all monkeys (Figure 7) but was not correlated with recovery on the mDB 2^nd^ well task.

**FIGURE 8:**
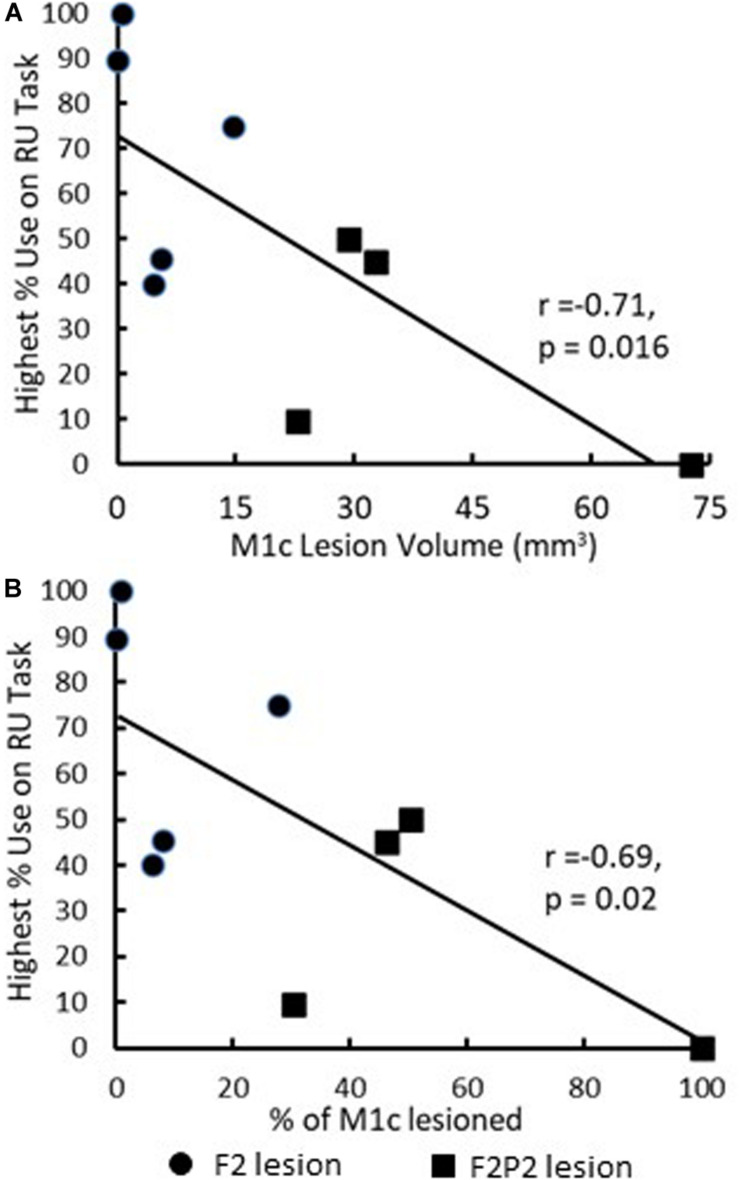
Scattergraphs showing the relationship between highest %use of the contralesional hand in a single RU test with M1c lesion volume **(A)** and percentage damage to M1c arm/hand area **(B)**. Each plotted point is data for a single monkey. All nine monkeys were included in this figure and correlation analysis.

Recovery in the RU task was closely associated only with volume and percentage of lesion to caudal M1 in both F2 and F2P2 lesion cases. There were no significant correlations of post-lesion week of first attempt or success with the contralesional hand in the RU task with total, gray or white matter lesion volumes (*p* > 0.085). Similarly, there were no associations of average %use of the contralesional hand in the RU task or highest %use in a single RU test with total, gray or white matter lesion volumes or lesion volumes of the frontal lobe (*p* > 0.1). However, highest %use of the contralesional hand in a single RU test was significantly inversely correlated with volume and percentage of lesion to M1c among all lesion cases (Figure 8, *p* < 0.02) but not with total frontal gray or white matter lesion volume (*p* > 0.219). Among the four F2P2 lesioned cases, there was no indication of a relationship between highest %use of the contralesional hand and volume or percentage of lesion to S1r (*p* > 0.25) or to parietal gray or white matter lesion (*p* > 0.14).

## Discussion

The observations described in this report provide new insight on the potential role of cerebral cortical injury to the development of reduced use and non-use of the contralesional hand. However, it is not clear whether the observed reduced use or non-use in a task allowing use of either hand is caused by impairment due to the lesion, neglect or other motivational factors. The current findings add to our previous report on reduced contralesional hand use in rhesus monkeys with isolated frontal lobe lesions (Darling et al., 2010), showing that up to 6 months of contralesional hand non-use can result following a combined lesion of frontal motor cortex and anterior parietal cortex, especially M1c and S1r. Occasional forced use of the contralesional hand for food acquisition during motor testing sessions was clearly sufficient to overcome or prevent non-use following most frontal and frontoparietal lesions. Of note, in our previous study only one monkey with a large frontal lobe lesion of arm areas of M1, LPMC and M2 (category F3 lesion – case SDM56 – gray and white matter lesion volumes of 407.54 mm^3^ and 64.01 mm^3^) and no parietal cortex injury demonstrated non-use of the contralesional hand for 6 months (i.e., never used the contralesional hand in the RU test). However, monkeys with even larger frontal lobe lesions including preSMA and prefrontal cortex exhibited non-use for only 2–4 weeks (see Table 2 of Darling et al., 2010). In our current study only one monkey (SDM91) with a much smaller total frontoparietal lesion volume (Table 1) but including 100% of both M1r and M1c along with LPMC and APC (including 69.9% of S1r) exhibited 4 months of severe non-use (never used the contralesional hand in any motor testing) despite some preserved capacity to execute fine hand and finger movements to successfully grasp and manipulate small food objects in the mDB and mMAP tasks during the first eight post-lesion weeks. After 8 weeks post-lesion SDM91 would not use the contralesional hand in either the mDB or mMAP tasks, thereby demonstrating non-use even when it was necessary to use that hand to successfully acquire small food targets. Yet the F3 lesioned monkey (SDM56) from the previously mentioned study also showed extended non-use in the RU task, but exhibited much better recovery in the mDB and mMAP tasks than SDM91 [i.e., consistent successful acquisition of food pellets in the mDB task by the 6^*th*^ post-lesion week and manipulation skill recovery of 0.5 on the best well mDB task for SDM56 – see Tables 2, 3 of Darling et al. (2010) vs. never being consistently successful in the mDB task and manipulation skill recovery of 0.05 by SDM91]. It therefore appears likely that complete or near complete lesions to both M1c and S1r, are major contributors to development of severe contralesional hand non-use whereas very large lesions limited to the frontal lobe do not induce such severe non-use of the contralesional hand. Thus, it appears that under these circumstances lesion location (i.e., to M1c and S1r) is more important than lesion volume in determining development of reduced use and non-use.

Additional support for a major contribution of M1c and S1r injury to RU or non-use comes from early work which showed that recovery of contralesional upper limb motor function after very large lateral motor cortex (M1 and LPMC) lesions in rhesus macaques required constraint of the ipsilesional (less impaired) limb and extensive forced use of the contralesional upper limb for recovery of fine hand/digit movements (Ogden and Franz, 1917). In their lesion procedure, Ogden and Franz ensured that motor cortex located deep in the central sulcus (M1c) was damaged by inserting a “white-hot cautery” 6–8 mm deep into its anterior bank “close to and parallel with the fissure.” It is likely that this surgical procedure also damaged part of the rostral postcentral gyrus and specifically, the primary somatosensory area (S1r) that is buried in the depths of the central sulcus. In fact, Ogden and Franz clearly indicated on their lesion map of one animal experiment (see their Figure 1), that the postcentral gyrus appeared “apparently abnormal” and further noted that “This area may have been involved because of changes in blood supply in the application of the cautery to the precentral cortex.” Indeed, inserting a cautery device into the central sulcus region of the monkey would likely ligate the Rolandic artery which directly supplies both M1c and S1r and surrounding cortex. Its ligation would almost certainly result in necrosis of both cortical areas. Importantly, as we verified histologically, S1r was not damaged in our F2 lesion cases of the present work, nor was most of M1c removed, and notably none of these monkeys exhibited severe non-use (Tables 1–3). When considering the above, the lesion procedure applied by Ogden and Franz clearly created a very severe lesion with longer duration of contralateral limb paralysis (26 days) than we observed in our cases (2–3 days), probably due to the combination of precentral, postcentral and subcortical white matter damage.

In contrast to the findings of Ogden and Franz (1917), Leyton and Sherrington (1917) reported that resection of the M1 arm/hand areas, including M1c, causes short-term RU but not non-use. Indeed, excising the M1 area “yielding primary movements of the thumb, index, fingers, wrist and elbow” and “the anterior wall of the centralis fissure” resulted in an apparently complete recovery of fine motor function within 1 month (Leyton and Sherrington, 1917). Similarly, reports from M1 lesion experiments in lemurs involving removal of motor cortex on one side “as completely as possible,” indicated that paresis following the lesion was similar to that in our cases as it passed “to a great extent” within 2 days and, by 14 days post-lesion, “it was difficult to recognize any paralysis”(Mott and Halliburton, 1908). Thus, findings from these early studies together with our current study would suggest that the rostral part of the postcentral gyrus (S1r) must be lesioned in addition to M1 damage, including M1c, to produce severe non-use of cortical origin.

Whether a pure postcentral gyrus lesion including the caudal bank of the central sulcus (S1r) might produce RU or non-use of the contralateral hand is an interesting question. As discussed in the Introduction, a pure sensory lesion by sectioning the dorsal roots for one upper limb can produce severe motor impairment and non-use in monkeys. Contrary to this observation, a behavioral study following pure postcentral gyrus lesions including the caudal bank of the central sulcus, with presumably its rostral bank spared, showed that monkeys would use the contralesional hand, albeit with poorer coordination of digit movements, in the acute recovery phase after the lesion (Kennard and Kessler, 1940). However, it is important to recognize that Kennard and Kessler (1940) did not provide histological verification of their lesions, thus the true extent of the S1r extirpation is unknown. In a more extensive and detailed study in *Macaca mulatta*, Peele reported similar behavioral observations following isolated postcentral gyrus lesions as the animals initially were “loathe to use the affected hand though they could when necessary” but by 14–21 days post-lesion “No lack of the desire to use the affected hand existed” despite slowness and some remaining ataxia (Peele, 1944). Importantly, in contrast to the Kennard and Kessler report, Pele performed a careful histological assessment of his parietal lesion cases and published this information in his 1942 paper on efferent cortical and subcortical parietal connections with the aid of the Marchi silver impregnation technique (Peele, 1942). His microscopic analysis of the postcentral lesions revealed complete removal of area 3 in the posterior bank of the central sulcus (see Peele, 1942- Plate IV) including what is now considered area 1 lining the dorsal most part of the posterior bank cortex (Morecraft et al., 2004). Thus, he presented three behavioral experiments (2 with a 21-day duration of observation and one with 381 days of observation) with complete and isolated removal of S1r with no lack of desire to use the contralesional hand by the third post-lesion week. Considering these classical observations, it can be concluded that acute or long-term non-use does not occur after pure postcentral gyrus lesions that include S1r.

As emphasized, the current observations, together with the early work summarized above, suggest that combined extensive lesions of peri-Rolandic primary motor and primary somatosensory processing areas are probably required to cause severe and lasting non-use of the contralesional hand. It is surprising, however, that SDM91 was able to use the contralesional hand successfully in larger wells of the mDB task and in the easier mMAP tasks (flat surface and straight rod) during the first eight post-lesion weeks before severe non-use developed. As mentioned above, this indicated that SDM91 had some preserved functional capacity to execute independent hand and finger movements after the injury, but eventually chose not to use the contralesional hand on the various motor tests. This delay in development of severe non-use may result from a progressive degradation/loss of the spared M2 corticospinal projection over this 8-week period in SDM91 as we observed that this monkey had substantially fewer terminal boutons in this projection to C5-T1 ventral horn (lamina IX) motoneurons than all the other F2P2 lesion cases, all F2 lesion cases and all controls (Morecraft et al., 2015). Other spared corticospinal projections could have additionally been negatively affected in this manner, such as those originating from the region of the cingulate motor cortex (Morecraft et al., 1997). Perhaps also contributing to severe non-use in SDM91 is that, unlike all other F2P2 cases which showed significant enhanced corticoreticular projections from spared M2, there was no evidence of an increased M2 corticoreticular projection to the medulla in SDM91 (Darling et al., 2018). Collectively the present behavioral observations and our recent neuroanatomical findings provide convincing evidence that in the absence of rehabilitative intervention severe long-term non-use results in deterioration of corticofugal projections from spared cortical motor areas (i.e., M2) including the corticospinal and corticoreticular projections in *Macaca mulatta* (Morecraft et al., 2015; Darling et al., 2018).

Motivation may have also played an important role in development of RU or non-use in our experiments. One of the F2P2 lesion cases (SDM83) used the contralesional hand to successfully acquire a food target in the RU task before being successful in the mDB task on any well, suggesting strong motivation to recover use of that hand probably due to limited food restriction and previous experience using that hand to obtain food rewards. Coupled with our previous finding that two monkeys with large lesions of the frontal lobe attempted to use the contralesional hand in the RU test before making an attempt in the mDB task clearly shows that high motivation to use the hand when permitted, in spite of motor deficits, plays a role in preventing RU following cortical injury (Darling et al., 2010). In this regard, it was surprising that SDM91 never attempted to use the contralesional hand in the RU task and developed severe non-use 8 weeks after the lesion despite clearly having some ability to use the contralesional hand including to grasp and manipulate pellets out of small wells on the mDB task (Morecraft et al., 2015; Darling et al., 2016). Perhaps extensive injury to postcentral sensory processing areas is a factor in causing neglect and reducing motivation to recover use of the more impaired hand, as well as having a detrimental effect on the descending projections from spared M2 (SMA) to motor nuclei in the brainstem (Darling et al., 2018) and spinal cord (Morecraft et al., 2015) that reduces motor recovery.

An important point to consider is whether reduced use or non-use of the contralateral limb is properly considered to be a learned phenomenon. The term LNU was initially used to characterize lack of use of a limb after dorsal rhizotomy blocked all afferent feedback from sensory receptors of one limb in rhesus monkeys. Because there was no direct damage to the descending connections to motor neurons or to motor structures of the CNS, there was no true paralysis of the limb and the non-use was assumed to be learned instead of being attributed to impairment due to the lesion. Lack of use of the limb despite the apparently intact motor system was attributed to initial clumsiness and failure to acquire desirable objects with the deafferented limb followed by success with the other hand reinforcing use of the intact limb for all motor tasks. An alternative interpretation is that non-use or reduced use of the deafferented limb is due to actual impairment rather than a learned suppression of its use. That is, deafferentation removes important synaptic inputs onto motor neurons that are probably necessary to control skillful limb movement (Ghez et al., 1995; Sainburg et al., 1995). Thus, the animal chooses not to use the deafferented limb unless forced to and is clumsy when forced. Extension of the LNU concept to stroke induced hemiparesis might also be considered inappropriate in that there is direct damage to the CNS motor areas or their projections affecting descending inputs onto motor neurons that are considered necessary especially for fine control of movements of the hand and digits (Lemon, 2019). Although forced use with constraint of the less impaired limb leads to some recovery of function after dorsal rhizotomy (Taub et al., 1977) and excellent recovery of function in rhesus monkeys with severe sensorimotor cortex damage (Ogden and Franz, 1917), none of these studies quantitatively evaluated hand use when the animal had choice of which hand to use. Our present work shows that non-use of the contralesional hand developed in such a task only in a monkey (SDM91) with near complete M1c and S1r lesions that exhibited very poor recovery of impaired hand motor function (Table 3). Thus, the non-use was probably due to impairment rather than learning. Interestingly, four F2 cases (SDM55, 70, 74, 80) and one F2P2 case recovered to pre-lesion skill or higher in at least one of the three fine motor tasks (Table 3). Three of those five cases had average and/or highest percentage use of the contralesional hand in the RU test that was comparable to or higher than in the pre-lesion handedness test (Table 3 – SDM55, SDM74, SDM80). We suggest that these monkeys did not exhibit a form of learned reduced use because they used the contralesional hand at a similar or higher rate than in the pre-lesion handedness test in some or many RU tests. In contrast, two cases had clearly lower average and highest percentage use of the contralesional hand in the RU test than in the pre-lesion handedness test despite very good recovery of its function (Table 3 – SDM70, SDM81). In our view, these may be considered a form of learned reduced use because contralesional (pre-lesion preferred) hand fine motor skill recovered very well, yet they primarily used the ipsilesional hand in the RU test.

We would like to make one final point, the longer time taken by F2P2 lesion cases to recover use of the contralesional hand in the RU task probably reflects their slower recovery of fine hand/digit motor function (Table 2 and Figures 4, 5). In this regard, it is important to note that the first attempt and success in the RU task do not reflect the data for case SDM91 since this monkey never chose to use the contralesional hand in the RU task despite some early attempts (4^*th*^ post-lesion week) and successful acquisitions (5^*th*^ post-lesion week) in the mDB task. Similarly, the level of recovery in the RU task, as indicated by the highest percentage of uses of the contralesional hand in a single RU test session (includes 0% use in the RU task for SDM91), probably reflects the level of recovery of fine hand motor function and some spared component of cortico-motoneuronal projections (e.g., perhaps from M2). However, it was curious that recovery in the RU task was correlated with recovery in the mMAP curved rod task, which is very different from the RU task, but not with recovery in the mDB best well or smaller well tasks which are similar to the RU task.

In conclusion, the present observations extend our previous findings (Darling et al., 2010) by showing that lesions of frontoparietal sensorimotor areas produce greater duration and severity of RU than lesions limited to M1 and LPMC. In addition, it appears that severe non-use results primarily from extensive lesion of M1c combined with S1r injury as we observed in SDM91. Overall, our findings, combined with those of previous studies in which there were large lesions of M1 including M1c while sparing S1r, suggest that both M1c and S1r have important roles in causing severe non-use for fine motor tasks (i.e., use of only the ipsilesional hand when either hand could be used to perform the task and lack of use of the contralesional hand when it must be used to successfully perform the task). The clear inverse relationship between highest %use of the contralesional hand in the RU task and M1c lesion volume (Figures 8A,B), in contrast to the lack of a clear relationship with parietal lesion volume, suggests that degree/volume of injury to M1c is the primary determinant of the extent of reduced use (when the monkey has a choice of which hand to use) due to the important role of cortico-motoneuronal neurons in precision grasping of small objects (Heffner and Masterton, 1975; Kuypers, 1981; Lemon and Griffiths, 2005; Schieber, 2007; Lemon, 2008, 2019). Consistent with this idea, it is known from early work that stimulation of the human peri-Rolandic area, especially just anterior to the central sulcus, primarily elicits movements of the contralateral arm, hand and digits, highlighting the role of this region in control of skilled upper limb movements (Penfield and Boldrey, 1937; Catani, 2017). Clinically, assessing the extent of peri-Rolandic injury in stroke patients may have prognostic value for: (1) predicting the risk potential for onset of non-use of the contralesional hand and long-term development of non-use and (2) planning rehabilitative intervention strategies to reduce or lower the risk of severe non-use. This may be particularly important in patients with extensive injury to the cortex lining the anterior and posterior banks of the central sulcus following middle cerebral artery cortical stroke.

## Data Availability Statement

The data that support the findings from this study are available upon reasonable request.

## Ethics Statement

The animal study was reviewed and approved by University of South Dakota Institutional Animal Care and Use Committee.

## Author Contributions

WD, RM, and MP designed the study. WD drafted the manuscript. WD, RM, and MP edited the manuscript. JG and KS-M collected the data. DR, MP, and WD analyzed the video and force data. JG, KS-M, and RM analyzed the lesion data. WD, MP, and RM interpreted the data. All authors approved the final manuscript.

## Conflict of Interest

The authors declare that the research was conducted in the absence of any commercial or financial relationships that could be construed as a potential conflict of interest.
